# Identification of Antithrombin-Modulating Genes. Role of *LARGE*, a Gene Encoding a Bifunctional Glycosyltransferase, in the Secretion of Proteins?

**DOI:** 10.1371/journal.pone.0064998

**Published:** 2013-05-21

**Authors:** María Eugenia de la Morena-Barrio, Alfonso Buil, Ana Isabel Antón, Irene Martínez-Martínez, Antonia Miñano, Ricardo Gutiérrez-Gallego, José Navarro-Fernández, Sonia Aguila, Juan Carlos Souto, Vicente Vicente, José Manuel Soria, Javier Corral

**Affiliations:** 1 Centro Regional de Hemodonación, Servicio de Hematología y Oncología Médica, HU Morales Meseguer, Regional Campus of International Excellence "Campus Mare Nostrum" University of Murcia, Murcia, Spain; 2 Unitat de Genòmica de Malalties Complexes, Institutd'Investigació Sant Pau (IIB-Sant), Barcelona, Spain; 3 Bio-analysis group, Neurosciences Research Program, IMIM Parc Salut Mar, PRBB, Barcelona, Spain; 4 Department of Experimental and Health Sciences, Pompeu Fabra University, PRBB, Barcelona, Spain; 5 Unitat d'Hemostasia i Trombosis. Institut d'Investigació Sant Pau (IIB-Sant), Barcelona, Spain; University of Bonn, Institut of experimental hematology and transfusion medicine, Germany

## Abstract

The haemostatic relevance of antithrombin together with the low genetic variability of *SERPINC1,* and the high heritability of plasma levels encourage the search for modulating genes. We used a hypothesis-free approach to identify these genes, evaluating associations between plasma antithrombin and 307,984 polymorphisms in the GAIT study (352 individuals from 21 Spanish families). Despite no SNP reaching the genome wide significance threshold, we verified milder positive associations in 307 blood donors from a different cohort. This validation study suggested *LARGE*, a gene encoding a protein with xylosyltransferase and glucuronyltransferase activities that forms heparin-like linear polysaccharides, as a potential modulator of antithrombin based on the significant association of one SNPs, rs762057, with anti-FXa activity, particularly after adjustment for age, sex and *SERPINC1* rs2227589 genotype, all factors influencing antithrombin levels (p = 0.02). Additional results sustained this association. *LARGE* silencing inHepG2 and HEK-EBNA cells did not affect *SERPINC1* mRNA levels but significantly reduced the secretion of antithrombin with moderate intracellular retention. Milder effects were observed on α1-antitrypsin, prothrombin and transferrin. Our study suggests LARGE as the first known modifier of plasma antithrombin, and proposes a new role for LARGE in modulating extracellular secretion of certain glycoproteins.

## Introduction

Antithrombin is an anticoagulant serpin essential for the haemostatic balance, as this molecule inhibits key procoagulant proteins, namely thrombin and FXa but also FIXa, FXIa, FXIIa and FVIIa [1], [2] by an extraordinary efficient suicide mechanism[3]. Consequently, complete antithrombin deficiency causes embryonic lethality and the heterozygous deficiency significantly increases (10–50 fold) the risk of thrombosis [4]. In general population the anti-FXa activity, the method widely used to diagnose antithrombin deficiency, shows a great variability with normal distribution [5]. Factors such as gender, body mass index, oral contraceptive intake or race seem to play a role in determining antithrombin levels [6]. Moreover, the high heritability of this trait (h = 0.486) sustains the role of genetic factors[7]. Indeed, the single nucleotide polymorphism (SNP), rs2227589, located in intron 1 of *SERPINC1*, the gene encoding antithrombin, showed significant association with antithrombin levels and explains up to 7% of antithrombin variability in the general population [8]. However, a recent study from our group showed a low genetic variability in *SERPINC1*, which plays minor influence in the inter-individual variability of antithrombin levels [9]. All these data suggest that other genes could indirectly modulate antithrombin levels.

Genome Wide Association Studies (GWAS) are the most popular and successful strategies for the identification of new susceptibility loci for multifactorial diseases [2], [10], although their relevance to identify new genetic risk factors for venous thrombosis has been recently questioned [11]. This methodology could give better results when used to identify genotype-phenotype associations [12]. Actually, this strategy has provided new and promising data concerning potential regulation of both levels of haemostatic factors or functions [13], [14].The objective of this work was to indentify modulating genes of antithrombin through a GWAS, sustaining any possitive association by additional experimental evidences.

## Materials and Methods

### Blood sampling, DNA purification and functional measurements

Blood was collected from the antecubital vein into citrate-tubes, and genomic DNA was purified. Platelet poor plasma was obtained within 5 min after blood collection, and stored at −70°C, prior to analysis. Plasma FXa-inhibiting activity was measured using a chromogenic method in presence of heparin (HaemosIL Liquid Antithrombin, Instrumentation Laboratory, Kirchheim, Germany) as previously reported [15]. Values were expressed as a percentage of the result observed in a control pool of citrated plasma from 100 healthy subjects (100%).

### Genome wide association study

We carried out a genotype-phenotype association study in the GAIT study, which included 352 individuals from 21 extended Spanish families [16]. Twelve of these families were selected on the basis of a proband with idiopathic thrombophilia, whereas the remaining 9 families were selected randomly. Average pedigree size was 19. Importantly, no family had congenital antithrombin deficiency.

A genome-wide set of 307,984 SNPs was typed in all of the participants using the Infinium® 317k Beadchip on the Illumina platform (San Diego, CA, USA). Genotype imputation was performed with Merlin [17] to avoid missing values and all genotypes were checked for Mendelian inconsistencies. In addition, any SNP with call rate<95%, minor allele frequency (MAF)<0.025 or failing to fit Hardy-Weinberg proportions taking into account multiple testing (p<5×10^−7^) was removed from the study. In total, 24,547 SNPs failed to pass the data cleaning criteria, leaving a set of 283,437 SNPs for further analysis.

### Validation study

A cohort of 307 Spanish Caucasian healthy blood donors (138 males/169 females), with a mean age of 43 years from a different region than the GAIT study was selected to replicate significant associations identified in the GWAS. Genotyping was performed using TaqMan® probes (Applied Biosystem, Madrid, Spain) specified in Table S1.

The Institutional Review Boards of the Hospital de la Santa Creu I Sant Pau (Barcelona, Spain) and Centro Regional de Hemodonación (Murcia, Spain) approved all protocols used in the GAIT and the replication cohort studies, and participants gave their informed written consent, in compliance with the Declaration of Helsinki, as amended in Edinburgh in 2000.

### Statistical analysis

In all studies, deviation from Hardy-Weinberg equilibrium (HWE) was investigated using a standard χ^2^ with 1 degree of freedom. In the GAIT study, HWE was tested using parental data only. In the GAIT study, association between SNPs and plasma anti-FXa activity was tested using a measured genotype association analysis assuming additive allele effects. This analysis was carried out using the variance-components methodology implemented in the SOLAR Version 4.0 software (Southwest Foundation for Biomedical Research, http://solar.sfbrgenetics.org/download.html) [18]. Analyses were adjusted for age, gender, and body mass index, and in females, for oral contraception as well.

Association between SNPs and plasma anti-FXa activity in the replication study was tested by a Student's *t*-test following a dominant model and adjusted for factors described to influence antithrombin levels (age, gender and the *SERPINC1*rs2227589 polymorphism) [6], [8]. This analysis was carried out using the Statistical Package for Social Science (SPSS version 15.0, USA). Haplotype analysis, association of haplotypes with anti-FXa activity and linkage disequilibrium analysis were calculated with the SNPstats software [19].

### *LARGE* and *SERPINC1* gene expression

*LARGE* gene expression was assessed in mononuclear cells of 10 healthy subjects by qRT-PCR using Hs00893935_m1 TaqMan® Gene Expression Assay (Applied Biosystem) and beta-actin (Hs99999903_m1) as constitutive reference gene.

*SERPINC1* gene expression in HepG2 and HEK-EBNA cell lines transfected with *LARGE* gene silencers was determined by qRT-PCR with SYBR® Green-Based Detection (Applied Biosystem) using Tubuline beta-2C chain as constitutive reference gene. Primers for amplification were: SERPINC1-F: TGTTCAGCCCTGAAAAGTCC; SERPINC1-R: GCTGCTTCACTGCCTTCTTC; TUBULINE-F: GGCCGGACAACTTCGTTT and TUBULINE-R: CCGCGCCTTCTGTGTAGT.

### Purification of plasma antithrombin. Proteomic and glycomic analysis

Plasma predominant antithrombin glycoform (α, with 4 *N*-glycans) from two subjects selected from 10 healthy blood donors, with the highest and lowest *LARGE* expression values, as well as antithrombin minor glycoform (β with 3 *N*-glycans) [20]from a pool of 100 healthy blood donors were purified by heparin affinity chromatography on HiTrap Heparin columns (GE Healthcare, Barcelona, Spain), using an ÄKTA Purifier (GE Healthcare) in 100 mM Tris-HCl and 10 mM citric acid, in a gradient from 0 to 0.25M NaCl and a step of 2M NaCl. Fractions with antithrombin were applied to a HiTrap Q column (GE Healthcare). Finally, proteins eluted were desalted through a dialysis tubing (Sigma Aldrich) and stored at −70°C, prior to analysis. The molecular mass and glucidic components of these molecules were determined by MALDI-TOF-MSanalysis and HILIC HPLC. Briefly,from protein molecular weight determination a solution of 3,5-dimethoxy-4-hydroxycinnamic acid (10 g/L) in acetonitrile (ACN)/water/trifluoroacetic acid (TFA) (50∶50∶0.1 by vol.) was employed. Experiments were carried out on a Voyager-DE™ STR Biospectrometry workstation (Applied Biosystems), equipped with a N_2_ laser (337 nm). Samples were measured both in the linear, providing information on the total number of different structures, and in the reflectron mode for identification of molecular formulas based on precise mass measurements. Recorded data were processed with Data Explorer™ Software (Applied Biosystems). The analysis of the *N*-glycans was performed by HILIC chromatography. Briefly, *N*-glycans were released with *N*-glycosidase F (Roche Diagnostics GmbH, Mannheim, Germany) following prior denaturing (5 min at 95°C in 150 mM sodium phosphate buffer, pH 7.4). Afterwards, samples were chilled on ice and digested with 0.6 U *N-*glycosidase F by incubation at 37°C, for 15 hours. Glycans were labeled as described (20) and subjected to chromatographic separation on an Agilent 1100 HPLC equipped with a fluorescence detector (1100 Agilent fluorescence module) using excitation and emission wavelengths of λ = 330 nm and λ = 420 nm, respectively. The following gradient conditions were employed on a ACQUITY UPLC™ BEH HILIC column (2.1×150 mm, 1.7 µm): solvent A was 10% 50 mM ammonium formate (pH 4.4) in 90% ACN, solvent B was 90% 50 mM ammonium formate (pH 4.4) in 10% ACN, and the flow rate was 15 µl/min. Following injection, samples were eluted by a linear gradient of 20–55% B over 100 min, followed by a linear gradient of 55–100% B over the next 5 min. The column was eluted using 100% B for 2 min, and subsequently re-equilibrated in 20% B before injection of the next sample. The system was calibrated in glucose units (GU) using a 2-aminobenzamide (2-AB)-labelled dextran hydrolysate. The total running time was 125 min [21]. Mass spectrometric analyses of 2-AB-labeled glycans were performed in 2,5-dihydroxybenzoic acid (DHB) matrix (10 mg/ml) in ACN∶H_2_O (50∶50 v/v). Typically, spectra of sialylated *N*-glycans were acquired in linear mode with negative polarity, and in neutral *N*-glycans reflectron mode and positive polarity. External calibration of the spectrometer was performed using a mixture of 2-AB-labelled glucose oligomers in the positive-ion mode and 2-AB-derivatised fetuin *N*-glycans in the negative mode. Recorded data were processed with Data Explorer TM Software (Applied Biosystems).

### *LARGE* gene silencing and effect on different proteins

For these experiments we used two cell lines expressing antithrombin: HepG2 with constitutive antithrombin expression, and Human Embryonic Kidney cells expressing the Epstein Barr Nuclear Antigen 1 (HEK-EBNA) transiently transfected with pCEP4-AT plasmid (generously provided by Prof. JA Huntington) that expressed high levels of the beta glycoform of human antithrombin [22]. HepG2 and HEK-EBNA cells were grown to 60% confluence at 37°C, 5% CO_2_, in DMEM (Invitrogen, Barcelona, Spain) supplemented with 5% fetal bovine serum (Sigma-Aldrich, Madrid, Spain). Then, they were transfected with 50 nM of specific *LARGE* siRNA: s17620 (Applied Biosystems) for 30 minutes in OptiMEM with siPORT™ (Applied Biosystem). Appropriate controls: transfections without siRNA, or with 50 nM of scramble siRNA (*Silencer*® Negative Control AM4611, Applied Biosystem) were used. After 12 hours, the cells were washed with PBS and exchanged into CD-CHO medium (Invitrogen) supplemented with 4 mM L-glutamine (Invitrogen). Cells were grown at 37°C for 48 hours. Then, RNA was purified using TRIzol® Reagent (Invitrogen) following manufacturer instructions. We determined the silencing efficiency evaluating *LARGE* and *SERPINC1* expression by qRT-PCR, as indicated above. Additionally, conditioned medium was harvested and in case of HepG2 cell cultures, concentrated 5-fold using a CentriVap Concentrator (Labconco, Kansas City, MO, USA). The levels of secreted antithrombin, transferrin, prothrombin and α1-antitripsin in conditioned medium were determined by western blotting, essentially as described elsewhere [23]. Briefly, electrophoresis was carried out using sodium dodecyl sulfate–polyacrylamide gel electrophoresis (SDS-PAGE) in 10% (w/v) polyacrylamide gels under reducing conditions. Proteins were transblotted onto a polyvinylidenedifluoride membrane. Proteins were immunostained with specific rabbit [anti-human antithrombin (Sigma Aldrich) and anti-human α1-antitripsin (Dako Diagnostics, Glostrup, Denmark)], goat [anti-human transferrin (Sigma Aldrich)], or sheep [anti-human prothrombin (Cerdalane laboratories, Burlington, Ontario, Canada)] polyclonal antibodies; followed by proper secondary IgG-horseradish peroxidase conjugates (GE Healthcare), and ECL detection (GE Healthcare). Antithrombin levels in the conditioned medium were also determined by a home-made ELISA, as previously described [23]. Additionally, anti-FXa activity of conditioned medium was measured by the chromogenic method described above. Finally, we also evaluated the intracellular content of antithrombin by western blotting and immunofluorescence, basically as previously described [23]. Briefly, cells were extensively washed with sterile PBS and then lysated with 50 µl of lysis buffer (10 mM TrisHCl, 0.5 mM DTT, 0.035% SDS, 1 mM EGTA, 50 mM sodium fluoride, 50 µM sodium orthovanadate, 5 mM benzamidine and 20 mM phenylmethylsulphonyl fluoride) and stored at −70°C, prior to analysis. Intracellular antithrombin was evaluated by Western blotting, essentially as indicated above. For immunofluorescence analysis, cells were fixed with an equal volume of 4% paraformaldehyde in PBS buffer pH 7.4 (22°C, 20 min). After fixation, cells were washed with PBS, permeabilized with 0.1% Saponin, 0.2% Gelatin, 0.02% Azide (3×5 min). All subsequent incubations and washes contained 0.1% Saponin, 0.2% Gelatin, 0.02% Azide in PBS buffer. Anti-antithrombin antibody was used at 1∶1000 and incubated for 1 h at 22°C. Indirect immunofluorescence was carried out using the appropriate fluorescein conjugated goat anti-Rabbit IgG (Vector laboratories, Burlingame, CA, USA) 1∶1000. Fluorescence was analyzed on a Confocal Microscope LEICA TCS-SP2 using its associated software (Leica Microsystems, Barcelona, Spain).

## Results

### GWAS analysis. Genotype-antithrombin levels associations in the GAIT study

**Figure 1:**
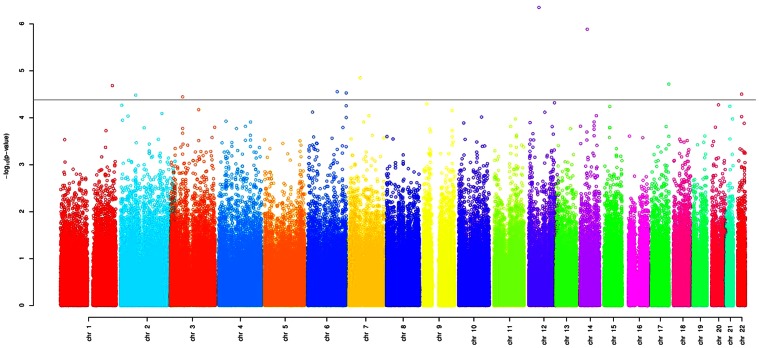
Manhattan plot GWAS with antithrombin phenotype. The thresholdof significance to select candidate SNPs for validation is also shown.

The plasma antithrombin levels, determined as anti-FXa activity, had a normal distribution in the GAIT study, with a medium value of 109.05% of the reference plasma and 154% and 78% as extreme values. No SNP was found associated with plasma anti-FXa activity at a genome-wide significance level (Figure 1). For validation analysis we selected the 10 SNPs with the strongest association (p<4×10E-05). Interestingly, 2 additional polymorphisms affecting *LARGE*, one of the gene identified, also showed significant association with anti-FXa activity, and were also selected to be validated (rs762057 and rs240082). Two of the *LARGE* SNPs (rs713703 and rs762057) displayed high linkage disequilibrium (D^2^ = 0.81). Table 1 displays the list of the polymorphisms, showing the p-value for the association with anti-FXa activity, the chromosomal location, and the gene potentially affected.

**Table 1:** Single nucleotide polymorphisms (SNPs) that associated with anti-FXa activity in the GWAS of the GAIT study and that were selected for validation studies.

SNPS	PVAL	BSNP	V-EXPL	CHR	LOC	GENE
rs10880942	0.00000044	−0.734718	0.081805	12	44905397	*SLC38A1*
rs2356895	0.00000128	−0.454476	0.088341	14	50898162	*LOC283553*
rs1860867	0.0000141	0.741308	0.050958	7	51762794	*COBL*
rs9896932	0.0000191	−1.361577	0.028271	17	77848326	*CD7*
rs1411771	0.0000206	0.361303	0.063374	1	230241398	*DISC1*
rs2152192	0.0000277	0.765150	0.061933	6	130510819	*SAMD3*
rs13193455	0.0000295	0.362923	0.029200	6	170354404	*LOC154449*
rs713703	0.0000313	0.330586	0.058187	22	32286080	*LARGE*
rs11681944	0.0000328	−0.338764	0.062070	2	68176083	*C1D*
rs6768189	0.0000357	0.429999	0.051721	3	54446050	*CACNA2D3*
rs762057	0.0000944	0.310900	0.052599	22	32280513	*LARGE*
rs240082	0.0010970	0.707098	0.039564	22	32428417	*LARGE*

### Validation study

The 10SNPs that showed stronger statistical association with anti-FXa in the GWAS, as well as the 2 additional *LARGE*SNPs were genotyped in 307 blood donors from a different Spanish region. Only rs762057maintained a significant association with anti-FXa levels in the validation cohort (p = 0.047) (Table 2). Multivariate analysis including age, gender and rs2227589, a SNP in *SERPINC1* gene previously reported to be associated with plasma anti-FXa activity [8], increased the significance between rs762057and anti-FXa activity (p = 0.02) (Table 2). Finally, *LARGE*haplotype analysis in the validation cohort revealed 5 frequent haplotypes, one of them (H2) significantly associated with anti-FXa activity (p = 0.030) (Table 3).

**Table 2:** Genotype-phenotype analysis in the validation study.

SNP	Gene	Genotype	Anti-FXa	Crude p	Adjusted p
1. rs10880942	*SLC38A1*	T/T	96.3±7.4		
		T/C+C/C	97.1±8.1	0.444	0.414
2. rs2356895	*LOC283553*	T/T	96.2±7.4		
		T/C+C/C	96.8±7.8	0.510	0.240
3. rs1860867	*COBL*	G/G	96.7±7.4		
		G/A+A/A	95.1±8.4	0.178	0.240
4. rs9896932	*CD7*	A/A	96.3±7.5		
		A/G+G/G	96.7±8.3	0.800	0.870
5. rs1411771	*DISC1*	T/T	95.7±7.9		
		T/C+C/C	97.0±7.2	0.122	0.078
6. rs2152192	*SAMD3*	G/G	96.4±7.6		
		G/A+A/A	96.2±7.4	0.839	0.998
7. rs13193455	*LOC154449*	G/G	96.2±7.5		
		G/A+A/A	96.6±7.6	0.651	0.705
8. rs762057	*LARGE*	G/G	95.3±6.9		
		G/A+A/A	97.1±7.7	0.047	0.020
9. rs11681944	*C1D*	G/G	96.3±8.0		
		G/A+A/A	96.6±7.1	0.730	0.655
10. rs6768189	*CACNA2D3*	G/G	96.8±7.8		
		G/A+A/A	95.6±6.9	0.158	0.198
11. rs713703	*LARGE*	T/T	95.2±7.2		
		T/C + C/C	96.8±7.5	0.082	0.087
12. rs240082	*LARGE*	A/A	96.4±7.7		
		A/G + G/G	96.0±6.9	0.800	0.733

**Table 3:** *LARGE* haplotypes identified in the validation study and their correlation with anti-FXa activity.

Haplotype	rs762057	rs713703	rs240082	Frequency	Difference (95% CI)	P
1	G	T	A	0.4523	0.00	—
2	A	C	A	0.403	1.4 (0.12–2.68)	0.033
3	G	C	A	0.0666	2.24 (−0.34–4.81)	0.09
4	A	T	A	0.0399	1.8 (−1.36–4.95)	0.27
5	G	T	G	0.0258	1.18 (−3.17–5.52)	0.6
rare	*	*	*	0.0124	−1.42 (−8.16–5.31)	0.68

### Functional studies

In order to verify the potential role of *LARGE* as a modulating gene of antithrombin further functional studies were performed.

**Figure 2:**
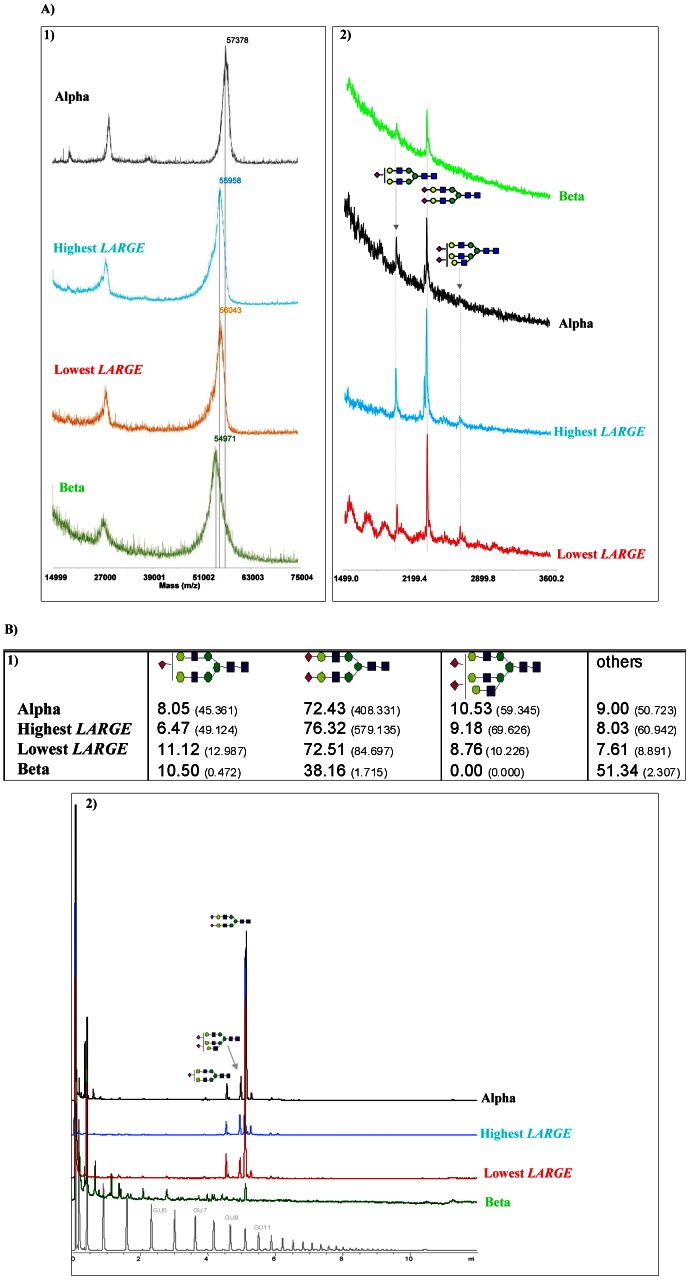
Glycomic and proteomic analysis of α-antithrombin purified from plasma of healthy subjects with the highest (blue) and lowest (red) *LARGE* expression. As controls we also used antithrombin glycoforms α (black), and β (green) purified from a pool of 100 healthy blood donors. The β glycoform has 3 *N*-glycans since it lacks N-glycosylation at N-135. A) MALDI TOF mass spectrometric analysis of: 1) Intact glycoproteins; 2) 2AB-labeled N-glycans. B) HPLC data. 1) Distribution of the glycan structures of antithrombin specimens. Values are represented as % of total glycan pool. Between brackets are the absolute fluorescence units. 2) HILIC HPLC profiles of antithrombin specimens.

Since *LARGE* codes an enzyme involved in post-translational glycosylation, and glycosylation of antithrombin plays a relevant role in the function of this serpin, particularly in the heparin affinity [20], [24], [25], our first hypothesis considered that differential expression or function of *LARGE* could result in distinct glycosylation of antithrombin. In order to verify this hypothesis, proteomic and glycomic studies were done with the main plasma antithrombin glycoform (α-antithrombin) purified from the subjects with the highest and lowest *LARGE* expression. However, their molecular masses were very similar, and glycomic studies showed fluctuations but not significant differences on the level or type of glucidic components (Figure 2). These results suggested that the association of *LARGE*with anti-FXa activity might be explained by a quantitative defect rather than by qualitative differences caused by the differential *LARGE* expression, but this can be questioned because of the weak expression of *LARGE* in mononuclear cells and the moderate differences found in healthy subjects with the highest and lowest *LARGE*expression (6.2-fold: 0.028 and 0.0045 units relatives to the expression of the constitutive gene, respectively).

**Figure 3:**
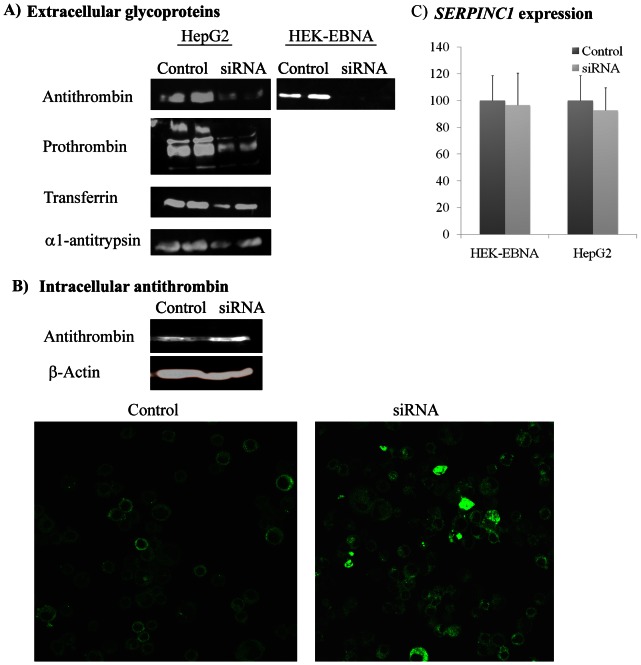
Consequences of *LARGE* gene silencing in HepG2 and HEK-EBNA cell lines. A) Secreted proteins to the conditioned medium evaluated by immunoblotting. B) Effect on intracellular antithrombin from HepG2 cells analyzed by immunofluorescence and immunoblotting. C) Effect on the levels of *SERPINC1* expression in HEK-EBNA and HepG2 cell lines. Immunoblots and immunofluorescence figures are representative of at least 3 independent experiments. Control represents cells transfected with scramble siRNA, although similar results were observed in cells transfected without siRNA.

To strongly sustain the relevance of *LARGE* on antithrombin levels, we carried out silencing experiments in HepG2 and HEK-EBNA cell lines. Secretion of antithrombin to the conditioned medium in both HepG2 was significantly reduced in silenced cells; 4-fold by western blot (Figure 3A) and 10-fold by ELISA (0.01±0.01 mg/ml compared to 0.15±0.20 mg/ml of control cells). The reduction was more significant in HEK-EBNA cells (Figure 3A). However,according to electrophoretic data, secreted antithrombin from silenced cells shows similar sizeto that of control cells(Figure 3A). Interestingly, anti-FXa activity in the conditioned medium of *LARGE*silenced cells was 59±30% and 11±12%of that found in control cells transfected with the scramble siRNA or without siRNA in HepG2 and HEK-EBNA respectively. The reduction of antithrombin secretion paralleled with a moderate intracellular retention of this serpin according to the immunofluorescence and western blot results (Figure 3B).

Moreover, in order to determine the mechanisms underlying the modulation of antithrombin by *LARGE,* we measured *SERPINC1* expression in these cells. Silencing of *LARGE* did not significantly modify *SERPINC1* mRNA levels (Figure 3C).

In HepG2 cells, we also studied the effect of *LARGE* silencing on other proteins: prothrombin, transferrin and other hepatic serpin: α1-antitrypsin. As shown in Figure 3A, silencing of *LARGE* also reduced the secretion of all other proteins evaluated, although antithrombin seemed to be the most affected.

## Discussion

Few modulating genes of haemostatic factors have been described so far [26]. The aim of this study was the search forantithrombin-modulating genes by a multi-stage approach, the same approach followed by a very recent study that extended the search to protein C and protein S [27]. The identification of modulating genes of key anticoagulants might help to identify new genetic risk factors for venous thrombosis.However, both studies failed to find SNPs associated with antithrombin levels at genome-wide significance. These negative results, despite the high heritability of antithrombin levels, strongly suggest that different elements with potentialmoderate effect might modulate the levels of this key anticoagulant and strength the requirement of additional approaches using different experimental studies, like those used in our study, to identify antithrombin-modulating genes. Thus, our replication analysis has evaluated 12 SNPs with milder association with anti-FXa activity in the GWAS. One SNP affecting *LARGE*, the rs762057,and particularly the *LARGE*H2 haplotype defined by rs762057, rs713703 and rs240082, associated with a modest increase of anti-FXa activity. The relevance of these three *LARGE* SNPs on the heritability of anti-FXa-levels was minor: 4.3%, 4.9% and 4.0% for rs762057, rs713703 and rs240082, respectively, but rose to 7.8% when considering the threeSNPs together.It is possible that other polymorphisms not included in the chip, haplotypes or rare mutations of *LARGE* might have stronger functional consequences, but this remains to be investigated. Unfortunately, the name of this gene reflects its length and genetic variability. *LARGE*expands more than 756,000 bpand contains more than 7,790 known polymorphisms, a size and genetic variability that make difficult to dissect the genetic architecture of this gene and to evaluate its potential functional and pathological relevance.

As the GWAS approach hardly identified *LARGE* as a candidateantithrombin-modulating gene, additional experimental evidences were required to sustain a potential role of *LARGE* on the indirectregulation of the levels of this anticoagulant. Thus silencing experiments confirmed a role for *LARGE* modulating antithrombin levels. Moreover, these resultsmay also open new mechanisms or pathways involved in the folding, secretion, function or clearance of this important anticoagulant, which may also be extrapolated to other homologous proteins. Additionally, our study also opens new attractive roles for LARGE, a protein largely unknown. LARGEplays a critical role in the biosynthesis of functional *O*-glycans, particularly of α-dystroglycan (α-DG) [28], although its over expression competes to modify GlcNAc terminals with Gal to generate the functional glycans not only in *O*-linked but also in *N*-glycans in α-DG[29] and could mediate phosphoryl glycosylation on *N*-linked glycans of non-α-DG proteins [30]. Finally, an excellent and recent study demonstrated that LARGE could act as a bifunctional glycosyltransferase, with both xylosyltransferase and glucuronyltransferase activities, which produced repeating units of [–3-xylose–α1,3–glucuronic acid-β1–] [31]. How could LARGE modulate antithrombin levels? Since reduced expression of *LARGE* did not affect the expression of *SERPINC1,* we can rule out an indirect role of LARGE on the transcriptional regulation of antithrombin. A direct effect on the glycomic features of antithrombin might also be discarded. The reduced expression of *LARGE* seems to down-regulate the secretion of antithrombin, without significant intracellular accumulation, probably reflecting a degradation of abnormal folding proteins [32]. These data togheter with the impaired secretion of other proteins (α1-antitrypsin, prothrombin or transferrin) observed under silencing of *LARGE* encouraged us to suggest a new function for LARGE in intracellular folding and/or secretion. The fact that the main affected protein among all tested is antithrombin, a protein with an heparin binding domain [33],together with the fact thatthe glycan produced by LARGE resembles heparin-heparan sulfate (HS) and chondroitin-dermatan sulfate (CS-DS) glycosaminoglycans (GAGs) [31], make attractive this hypothesis. Further studies are required to verify this hypothesis and to define the exact mechanism involving LARGE on the folding, secretion and degradation pathways of glycoproteins, particularly antithrombin, and to determine the final effect on the haemostatic equilibrium, as LARGE might also reduce the secretion of prothrombotic proteins such as prothrombin.

## Supporting Information

Table S1 TaqMan® probes used for genotyping in the validation study.
(DOCX)
