# Supplementary material for: Identification of Antithrombin-Modulating Genes. Role of LARGE, a Gene Encoding a Bifunctional Glycosyltransferase, in the Secretion of Proteins?
Source: PLoS One. 2013 May 21;8(5):e64998. doi: 10.1371/journal.pone.0064998 (PMC3660365; doi:10.1371/journal.pone.0064998)
Supplement: Table S1 — TaqMan® probes used for genotyping in the validation study. (DOCX) [file pone.0064998.s001.docx]

**Table S1. TaqMan® probes used for genotyping in the validation study.**

| **Probe** | **SNP** | **Gene** |
| --- | --- | --- |
| **c_15834925_20** | rs2152192 | *SAMD3* |
| **c_2747618_20** | rs1411771 | *DISC1* |
| **c_9839576_10** | rs13193455 | *LOC 154449* |
| **c_1282266_10** | rs11681944 | *C1D* |
| **c_1298059_10** | rs6768189 | *CACNA2D3* |
| **c_ 30869706_10** | rs10880942 | *SLC38A1* |
| **c_11634765_10** | rs1860867 | *COBL* |
| **c_16207835_20** | rs2356895 | *LOC283553* |
| **c_30230995_10** | rs9896932 | *CD7* |
| **c_2486441_10** | rs762057 | *LARGE* |
| **c_1839936_10** | rs713703 | *LARGE* |
| **c_790509_10** | rs240082 | *LARGE* |
